# Training School

**Published:** 1876-01

**Authors:** 


### TRAINING SCHOOL.

We have seen an announcement in the public papers that it was in contemplation to establish somewhere in Pennsylvania, "*A Training School for Clergymen.*" As none was given, we are thrown upon our own resources for an explanation. As clergymen have had a scientific training at college, and a theological training in the seminary, and a moral and religious training from infancy by parents, Sunday School teaching and ministerial culture; and as morality, piety and learning would seem to cover the whole ground, we are in a quandary still. But as giving up never did any good, when a laudable object was in view, and as our Episcopal friends have not vouchsafed an enlightenment, we are disposed to ferret out the idea. From early and long and wide association with clergymen, and a high estimate of their character, their usefulness and their importance as to the true and permanent prosperity of any country, we will venture an opinion as to what they ought to be trained to, as supplementary to morality, piety and scholarship.

To our mind, the most impressive defect in that noble army of thirty thousand men who have consecrated themselves to the best interests of our nation, for a compensation not enough to feed and clothe them in a manner commensurate with their high office, and their due, is *a want of sympathy with men*, and next to that, *a want of the knowledge of human nature.* According to the present system, a minister becomes almost gray-headed before he fairly gets into the traces of an easy and profitable working. In reaching this point, there is many a trip and slip and fall, requiring sometimes long years to regain the standing, and sometimes it is regained—never! not from actual criminality, but inadvertence. All know how the young are apt to become enthusiastic in what they have confidence, especially when it becomes their bread. To be enthusiastic is to be in danger anywhere.

It was not until he *began to be about thirty years of age* that the Divine Redeemer entered fully on his ministry. From youth up to that time he worked at the trade of a carpenter, as did his reputed father before him. And if, with all the advantages of inspiration, it was necessary for him, to qualify himself fully for his office, that he should spend so long an apprenticeship with the people, may we not judge that it does possess high advantages for a man to mingle largely and long with men in their every-day callings and ambitions, in aiding him to find out what human nature is. The disciples and apostles were bred to secular callings, and we all know that he can best lead and control men who has the best key to the human heart.

Patrick Henry associated with all men; he even is reported by Thomas Jefferson, to have had rather a preference for low associations; and from knowledge here acquired, he derived his power.

It is comparatively a rare thing for a clergyman to become derelict; thus it is, that when such is the case, it has so startling an effect on a community. The very rarity increases its bad influences. We propose it, then, as a matter of reflection, which may lead to useful results—If a man never entered the work of the ministry until of an age and experience which would prevent any false step in any direction, and insure always the best steps to be taken towards securing any desired result, and were to work thus for fifteen years, would not larger and higher results be attained by ministerial efforts, than are reached under present circumstances? In other words, would not the ministry work much more efficiently in all directions, if each one, on entering it, had a better knowledge of human nature, and could more intimately enter into the sympathies of men, than they now do. And is there any better school for such objects than a long novitiate, than close associations with men in the daily pursuits of life? The Doctors certainly know that the thing which brings most discredit upon the profession is the haste of students to "*realize*," to be making money; entering the practice of medicine almost wholly upon a theoretic knowledge. Hence few attain eminence, and the fewest of the few obtain a fortune in the pursuit of their calling. And even in cases—the very rare cases—where young clergymen steer clear of quicksand and shoal and sunken rock, it is accomplished at such an effort, that health is made a wreck of, and they enter the haven of full usefulness, only in time to die, long, long before *the heat of the day comes on.* Under the whole view of the case, therefore, we believe that a five years' colportage on foot, with a bible and concordance, selling religious publications *at a per centage*, is the best theological seminary in the world; securing, as it would, at one and the same time, health, tact, knowledge, and piety.

We have said that it has occurred to us that clergymen are deficient in popular sympathy. They do not *make that allowance* for the short-comings of their fellows, which a true knowledge of the trials, hardships, discouragements and temptations of life would lead to. A good heart grows mellow as it approaches the grave. Old clergymen grow forbearing as they near heaven. We contend, if they could start with the largest share of this, many hearts would be won to religion that are never won at all. Harshness wrecks and wins not the inquiring soul.

COME UNTO ME, ALL YE THAT ARE WEARY AND HEAVY LADEN AND I WILL GIVE YOU REST, is the embodiment of heaven's sympathy, and it came from the lips of the Master. But who does not know how often the young clergyman, and the ignorant, hurls whole avalanches of incandescent brimstone at weary and worn humanity: even in the ruder times of Old Testament history, the Almighty is represented as "*winking*" at the ignorance of men. It seems to us that modern preaching is more imprecatory than deprecatory. "*If you don't you'll be damned*," instead of "*go and sin no more;*" instead of

"COME TO JESUS! SINNER, COME."

That we may not be mistaken in our meaning, we repeat, If our ministers, already better than we deserve, are to have additional training, let it be in the knowledge of human nature and human need; let them have a longer novitiate; let them have a term of close association with men in the active and healthful callings of human life, preparatory to entering the full ministry.

We did not say, without some hesitation, that there was a lack of sympathy with the people among clergymen, but an item has come to our knowledge since, which shows that ministers themselves have noticed something of the kind; for one of them, in accounting for his superior success, quaintly writes, "When you go a-fishing, my brother, you get a great hoop-pole for a handle, attach a large cod line and a big hook, with twice as much bait as the fish can swallow. Thus accoutred, you dash to the brook, throw in your line with a '*There! bite, you dogs.*' But with a little pole and a small line and a suitable bait, I creep up, slip them in, and *twitch 'em out*, until my basket is full."

